# A genome-wide association study on photic sneeze reflex in the Chinese population

**DOI:** 10.1038/s41598-019-41551-0

**Published:** 2019-03-21

**Authors:** Mengqiao Wang, Xinghan Sun, Yang Shi, Xiaojun Song, Hao Mi

**Affiliations:** 1 Department of Epidemiology and Biostatistics, West China School of Public Health, Sichuan University, Renmin South Road 16, Chengdu, Sichuan Province 610041 P.R. China; 2 Chengdu 23Mofang Biotechnology Co., Ltd., High-tech District E6-10, Chengdu, Sichuan Province 610042 P.R. China

## Abstract

Photic sneeze reflex (PSR) is an interesting but yet mysterious phenotype featured by individuals’ response of sneezing in exposure to bright light. To uncover the underlying genetic markers (single nucleotide polymorphisms, SNPs), a genome-wide association study (GWAS) was conducted exclusively in a Chinese population of 3417 individuals (PSR prevalence at 25.6%), and reproducibly identified both a replicative rs10427255 on 2q22.3 and a novel locus of rs1032507 on 3p12.1 in various effect models (additive, as well as dominant and recessive). Minor alleles respectively contributed to increased or reduced risk for PSR with odds ratio (95% confidence interval) at 1.68 ([1.50, 1.88]) for rs10427255 and 0.65 ([0.58, 0.72]) for rs1032507. The two independent SNPs were intergenic, and collectively enhanced PSR classification by lifting the area-under-curve value in ROC curve to 0.657. Together with previous GWAS in other populations, the result substantiated the polygenic and non-ethnicity-specific nature behind the PSR phenotype.

## Introduction

In comparison to Mendelian phenotypes that are exclusively explained by genetic variation at a single defined locus (generally in the coding region of a certain gene), complex phenotypes are caused by a collective effect of many genetic variants throughout the genome^1^, requiring “omics” studies to fine map the associated and even better the causal loci^2^. Such phenotypes include many challenging diseases (eg. Alzheimer’s, Parkinson’s, asthma etc.) as well as a great number of common and interesting traits (eg. height, chin dimple, age at menarche etc.)^3^, of which the underlying genetic mechanisms remain largely unelucidated. A genome-wide association study (GWAS) is a modern high-throughput method simultaneously genotyping up to 1 million common genetic variants (single nucleotide polymorphisms, or SNPs) with a gene chip, and scanning on a genome-wide level the significant association of hit SNPs with phenotypes-of-interest. Since its landmark achievement identifying loci associated with macular degeneration^4^, GWAS has thrived in the past decade^5–7^, and has been nonstop uncovering novel genetic loci strongly associated with many important diseases^8–10^ and interesting traits^11–13^.

Photic sneeze reflex (PSR), also known as autosomal dominant compulsive helio-ophthalmic outbursts (ACHOO) syndrome^14^, refers to uncontrollable reflexive sneezing in response to sudden exposure to bright light. Sneezing is generally a protective reflex expelling particles and irritants from the nasal cavity, but it is a puzzle how bright light could stimulate the sneeze reflex, and evolutionarily speaking, whether it has any physiological relevance. First reported in medical literature as early as in 1954^15^, PSR was a common but poorly understood phenotype with relatively high prevalence in diverse populations (on average affecting about one in every four people)^16^. Although the phenotype is not deleterious enough to qualify as a disease, PSR presents annoyance or even danger to susceptible people when they emerge from darkness or dim light into considerable brightness, such as drivers exiting a tunnel, pilots conducting flights, patients undergoing surgery, miners working underground etc. Study of military medicine revealed that PSR was not associated with specific wavelengths of light so the use of filtering lenses in sunglasses or goggles would not be helpful in mitigating the reflex^17^. Instead, PSR was mediated and induced by changes in the intensity of light^17^. Efforts to test or reproduce the reflex response in clinical setting by exposing individuals to bright light were unreliable, so in the absence of a standardized testing method, PSR was likely best diagnosed by directly questioning the individuals and identifying the affected ones by their self-reporting. Indeed, PSR-positive individuals are generally well aware of their reflex, while PSR-negative individuals are usually unaware or have even never heard of this reflex.

PSR has been known for long to have an underlying although unclear genetic basis. With pedigree study of a single index patient (self, father, brother and daughter being PSR-positive, but mother and wife being PSR-negative), PSR was likely under an autosomal dominant mode of inheritance^18^. However, that pedigree study was of limited sample size (only one family) so the underlying mode of inheritance for PSR likely remained inconclusive. It is natural to understand the blink of eyes in response to bright light but it remains hard to imagine the sneeze of nose to the same stimuli. Assuming an unidentified mediator between the eyes and the nose with regard to PSR, a potential candidate could be the trigeminal nerves, which are the largest cranial nerves mediating a known reflex of lacrimation in response to a punch to the nose^19^. However, in comparison of the photic reflex to the physical reflex, the unknown mechanism behind PSR in the central nervous system is likely more complex and mysterious than lacrimation given that light is unable to directly excite the trigeminal nerves^19^. In one study, cystinosis (a form of corneal abnormality) was reportedly associated with enhanced PSR in a patient^20^. Nevertheless, the biomedical mechanism of PSR remains largely unknown to this date.

PSR was likely a complex rather than Mendelian phenotype, and it may potentially be unique to humans or just impossible to study in non-human model organisms. To address the challenge, a few large-size GWAS were conducted to explore the association between genetic variants and PSR phenotype. A landmark study of 5390 participants in the U.S. identified a novel genome-wide significant SNP of rs10427255 at 2q22.3 locus (intergenic, with −log_10_(P) of 10.93 and OR of 1.32) and a candidate SNP of rs11856995 at 15q26.2 locus (intergenic, with −log_10_(P) of 7.13 and OR of 0.78)^21^. A similar GWAS analyzed 11409 Japanese individuals, and discovered genome-wide significant SNPs of rs1691483 and rs1694933 at 3p12.1 locus^22^. In addition, a GWAS of PSR on 99695 European-descendant individuals identified 50 strongly associated loci thanks to the enhanced statistical power conferred by its much larger sample size^3^. These GWAS differed in aspects such as gene chip used, sample size achieved, analytical methods applied etc. but most importantly in their target populations. It is of interest if PSR has any genetic association unique to different ethnicities. To our knowledge, no large-size GWAS on PSR has ever been conducted or published for the Chinese population, and we aim to investigate this issue to better reveal the genetics associated with this interesting phenotype.

## Results

### A large-sample study of photic sneeze reflex in the Chinese population

A sample of 3417 Chinese individuals were analyzed for the association between 419093 genetic variants and the PSR phenotype (Table S1). 874 participants self-reported as PSR-positive, and the corresponding prevalence of 25.6% (95% CI of [24.1%, 27.1%]) was similarly comparable to previously reported PSR prevalence in several western countries^16,23,24^. We observed a male prevalence of 30.1% (95% CI of [27.9%, 32.3%]) and a female prevalence of 21.1% (95% CI of [19.2%, 23.2%]), which were statistically different (*P* < 1 × 10^−8^). The sample was balanced regarding sex with 1700 males and 1717 females (*P* = 0.78), and no significant differences were observed in age distribution either between sexes (Fig. S2A) or between case-control groups (Fig. S2B). The aiming population was exclusively defined so all individuals recruited were Chinese citizens. In addition, principal component analysis (PCA) facilitated the classification of all participants into ancestry groups given their genetic variants. Scatter plots with the top principal components (PCs) revealed the clustering of most individuals into a single cohort and the limited range for diverting points (Fig. S3A), suggesting robust homogeneity of Chinese ancestry in the sample. Moreover, the top 10 PCs cumulatively explained over 55% of total genetic variation (Fig. S3B), and thus would be included in later statistical analysis as covariates to minimize any remaining genetic substructure.

### GWAS identified both replicative and novel genetic loci

**Figure 1:**
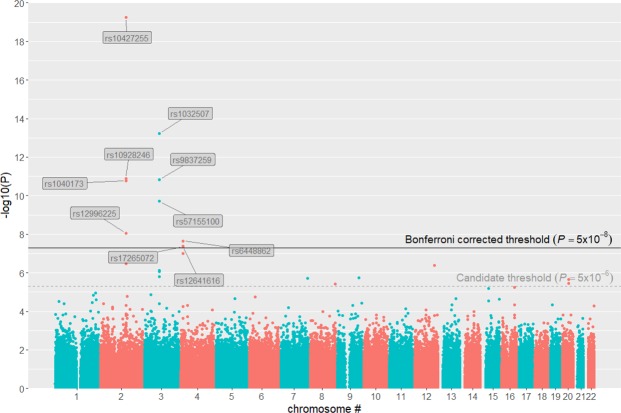
Manhattan plot of GWAS based on an additive effect model. Bonferroni corrected threshold and candidate threshold respectively correspond to 7.30 and 5.30 with regard to −log_10_(P).

**Figure 2:**
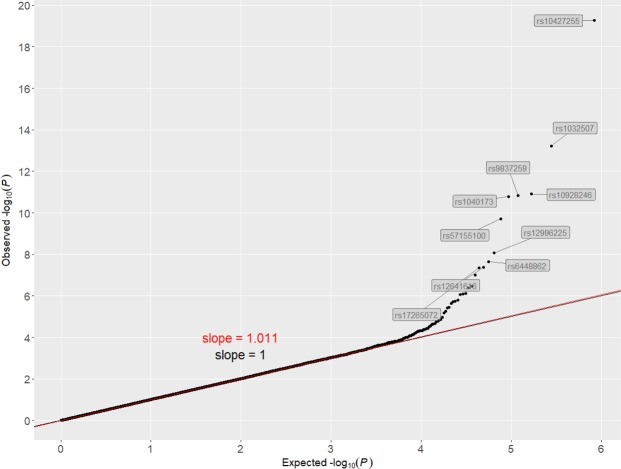
Quantile-quantile plot of observed *vs*. expected −log_10_(P) scores in GWAS.

**Figure 3:**
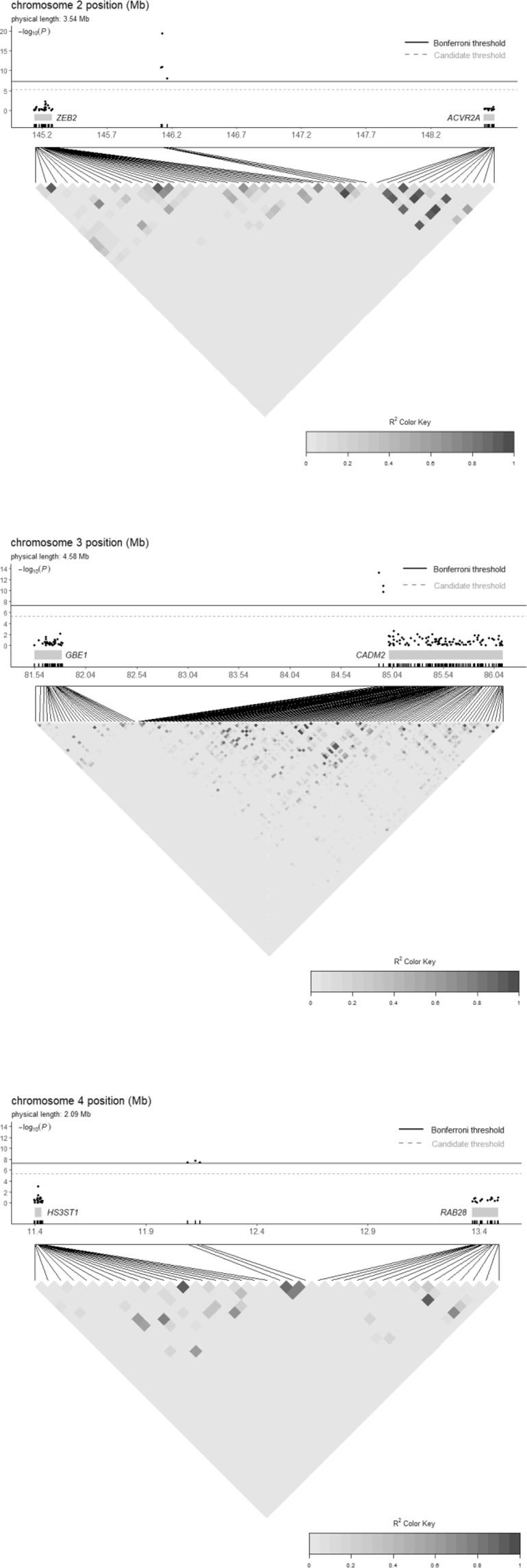
Association plot of hit SNPs to SNPs within their nearest upstream and downstream genes. For each gene, SNPs within the region of 5000 bp upstream of start codon and 5000 bp downstream of stop codon were included.

Based on an additive effect model and with a stringent Bonferroni-corrected threshold, GWAS identified 10 hit SNPs associated with PSR in 3 independent autosomal regions (Fig. 1 and Table 1). Given different PSR prevalence between two sexes in our sample, we also performed a sex-separated analysis and identified similar hit SNPs although the smaller sample size led to reduced statistical power and thus to the significance of some hit SNPs falling below the Bonferroni-corrected threshold (Fig. S4A for males and Fig. S4B for females). As quality control, SNP-level scores fell onto a line with the slope of unstandardized λ at 1.011, with no major shift from the line of *y* = *x* and thus without systematic bias (Fig. 2). For the additive effect model, the top hit in each region was respectively rs10427255 on chromosome 2, rs1032507 on chromosome 3, and rs6448862 on chromosome 4, to which the other 7 hit SNPs located closely adjacent and scored significant due to high linkage disequilibrium (Fig. S5). All hit SNPs were intergenic rather than within the open reading frame of any genes, thus unlikely to contribute to the PSR phenotype by directly modifying the structure or function of any potential proteins (Fig. 3). Among the 3 top regional hit SNPs, rs10427255 was 848 kb downstream of *ZEB2* (Zinc Finger E-Box Binding Homeobox 2) and 2477 kb upstream of *ACVR2A* (Activin A Receptor Type 2 A); rs1032507 was 3100 kb downstream of *GBE1* (1,4-Alpha-Glucan Branching Enzyme 1) and 97 kb upstream of *CADM2* (Cell Adhesion Molecule 2); rs6448862 was 694 kb downstream of *HS3ST1* (Heparan Sulfate-Glucosamine 3-Sulfotransferase 1) and 1245 kb upstream of *RAB28* (Member RAS Oncogene Family). Most of these hit SNPs were without substantial linkage to any measured SNPs within their neighbor genes (r^2^ < 0.005, Fig. 3), and the only exception appeared to be rs1032507 with mild linkage to a few SNPs in the initial coding region of *CADM2* (0.1 < r^2^ < 0.2, Fig. S6). Potential explanations of the observed associations could be 1). SNPs in promoter region could regulate gene expression (e.g. rs1032507 was possibly in the promoter of *CADM2*); 2). SNPs could affect phenotype through distal regulation so the nearest genes to the hit variants may often not be the responsibly causal ones; 3). SNPs may influence local epigenetic modifications and thus be indirectly associated with the phenotype. Nevertheless, GWAS identified 3 independent genetic variants significantly associated with PSR, but they did not directly affect the coding of particular genes, and thus the biology of PSR remained mechanistically unclear.

**Table 1:** List of 10 hit SNPs in GWAS based on the additive effect model.

#	SNP	Chr.	Position	Calls Call-rate		RA	RAF	MA	MAF	P(AA)	P(AB)	P(BB)	P value	−log_10_(P)
1	rs10427255	II	146125523	3417	1	C	0.56	T	0.44	0.20	0.48	0.31	5.52 × 10^−20^	19.26
2	rs1032507	III	84911291	3413	0.998	G	0.50	A	0.50	0.24	0.51	0.25	5.92 × 10^−14^	13.23
3	rs10928246	II	146129264	3409	0.997	A	0.57	G	0.43	0.19	0.49	0.33	1.27 × 10^−11^	10.90
4	rs9837259	III	84952678	3408	0.997	T	0.62	C	0.38	0.14	0.48	0.38	1.46 × 10^−11^	10.84
5	rs1040173	II	146120511	3416	0.999	T	0.57	C	0.43	0.19	0.48	0.33	1.64 × 10^−11^	10.78
6	rs57155100	III	84952298	3414	0.999	G	0.68	A	0.32	0.10	0.44	0.46	1.97 × 10^−10^	9.71
7	rs12996225	II	146166521	3374	0.987	T	0.63	C	0.37	0.14	0.46	0.40	8.73 × 10^−9^	8.06
8	rs6448862	IV	12124570	3327	0.973	C	0.67	T	0.33	0.10	0.46	0.44	2.23 × 10^−8^	7.65
9	rs12641616	IV	12088964	3415	0.999	A	0.67	G	0.33	0.11	0.45	0.44	4.30 × 10^−8^	7.37
10	rs17265072	IV	12143728	3407	0.997	T	0.64	C	0.36	0.13	0.46	0.41	4.37 × 10^−8^	7.36

RA - reference allele; RAF - reference allele frequency; MA - minor allele; MAF - minor allele frequency.

AA: genotype homozygous for minor allele; AB: genotype heterozygous; BB: genotype homozygous for reference allele.

Bonferroni corrected threshold (5 × 10^−8^) corresponds to 7.30 with regard to −log_10_(P).

PSR was reported as a likely autosomal dominant phenotype^18^, although the single-family-based evidence was not conclusive. Given its unknown mode of inheritance, in addition to the above additive effect model, we applied the same dataset to GWAS based on models of dominant effect or recessive effect. The same 4 hit SNPs on chromosome 2 and the same 3 hit SNPs on chromosome 3 remained significant in the dominant effect model (Fig. S7A); only rs10427255 on chromosome 2 and rs1032507 on chromosome 3 remained significant in the recessive effect model (Fig. S7B). The above results combined suggested that 2 independent genetic variants (rs10427255 and rs1032507) were robustly associated with PSR regardless of the effect models, but rs6448862 was only significant in the additive effect model. In all three models, rs10427255 scored a higher significance of association than rs1032507.

### rs10427255 and rs1032507 display independent and combinational associations with PSR

**Figure 4:**
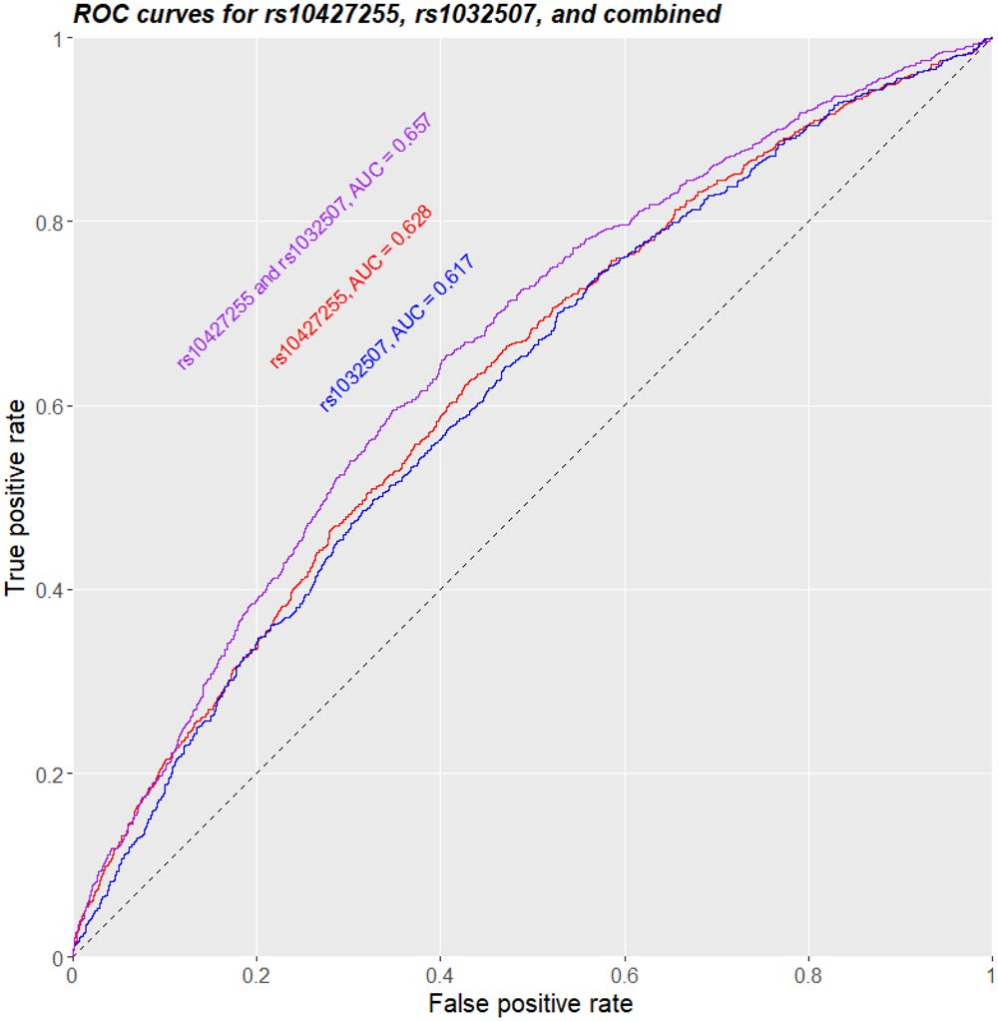
ROC curves for predicting PSR phenotype using rs10427255 alone (red), rs1032507 alone (blue), or rs10427255 and rs1032507 combined (purple).

With assumption of the additive effect model, rs10427255 had an odds ratio (OR) of 1.68 so each copy of minor allele (T) conferred a multiplicative 68% higher odds for PSR; in comparison, rs1032507 had an OR of 0.65 so each copy of minor allele (A) conferred a multiplicative 35% lower odds for PSR (Table 2). The effects of both SNPs should be independent given their locations on different chromosomes. Indeed, when combined together into the additive effect model, OR levels for both SNPs were largely unchanged compared to those in models with single SNP (Table 2). Regarding the ROC curve and the AUC value used for classification, rs10427255 displayed slightly better performance than rs1032507 (AUC of 0.628 *vs*. 0.617, Fig. 4), consistent with its higher association significance; interestingly, when combined, the 2 SNPs together demonstrated a substantial lift in PSR classification (AUC of 0.657, Fig. 4) compared to single SNP (bootstrap-based AUC test: P = 0.04 for combined *vs*. rs10427255 alone, and P = 2.3 × 10^−6^ for combined *vs*. rs1032507 alone), further articulating their independent but yet combinational associations with the PSR phenotype. Such phenomenon was also observed in both dominant effect model (Fig. S8A) and recessive effect model (Fig. S8B).

**Table 2:** Summary of hit SNPs: rs10427255, rs1032507, and both combined.

SNP	β	P value	−log_10_(P)	OR	95% CI_OR_	AUC
rs10427255	0.52	5.52 × 10^−20^	19.26	1.68	[1.50, 1.88]	0.628
rs1032507	−0.44	5.92 × 10^−14^	13.23	0.65	[0.58, 0.72]	0.617
rs10427255	0.53	3.37 × 10^−20^	19.47	1.69	[1.52, 1.90]	0.657
and rs1032507	−0.45	2.87 × 10^−14^	13.54	0.64	[0.57, 0.72]	

β: coefficient for SNP in the logistic-regression-based additive effect model.

OR: odds ratio for one copy of the minor allele of SNP.

95% CI_OR_: 95% confidence interval of OR.

AUC: area under the ROC curve.

**Figure 5:**
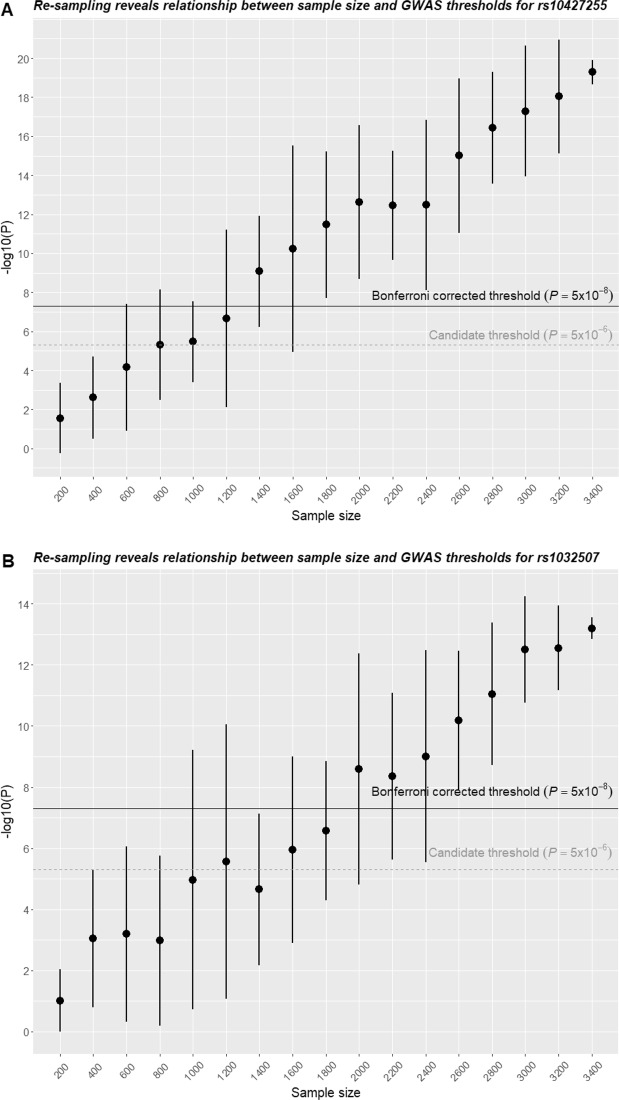
Sensitivity of hit SNPs to sample size. The total 3417 participants were repeatedly (10 times) resampled for an increment of 200 in sample size; average and 95% confidence interval for the significance score of −log_10_(P) were shown as points and vertical lines for hit SNPs of rs10427255 (**A**) and rs1032507 (**B**).

Just as large sample size generally confers higher statistical power, the number of study participants is an influential factor for GWAS and serves as an indirect manifestation for the association strength between the hit SNPs and the phenotype-of-interest^25,26^. By setting the cohort of 3417 individuals as the population and maintaining the prevalence of PSR as observed, we conducted repeated random re-sampling to obtain subsets of different sizes and accordingly evaluated the association significance between PSR and hit SNPs (rs10427255 and rs1032507). All repeated trials were likely to score genome-wide significance with size over 1800 for rs10427255 and size over 2600 for rs1032507, further arguing for the underlying strong associations (Fig. 5). Undoubtedly, these two associated genetic variants were unlikely to be the causal ones and it still remained a challenge to reveal the genetic and physiological mechanisms behind PSR. However, GWAS successfully identified rs10427255 and rs1032507 as independent polygenic risk predictors for PSR in the Chinese population, not only proving the values in self-reported phenotypic studies but also navigating future research to the defined sites and regions within the genome.

## Discussion

In our GWAS with a large sample of the Chinese population, two hit SNPs of rs10427255 and rs1032507 were associated with the PSR in genome-wide significance. With PSR prevalence comparable in the U.S. population (32.4%), rs10427255 was identified in a previous GWAS^21^, and its current replicative discovery in the Chinese population suggested a likely common marker for this phenotype across different ethnicities. Paradoxically, the minor allele of rs10427255 was switched in the U.S. population (C, minor allele frequency or MAF of 46%) *vs*. the Chinese populations (T, MAF of 44%), but in both populations, the minor allele contributed to significantly higher risk of PSR in the additive effect model. In comparison, the suggestively significant SNP of rs11856995 in the U.S. study scored low with −log_10_(P) of 2.8 in our study. The SNP of rs1032507 is a novel genetic variant with PSR and was not identified in the U.S. population. Another GWAS on PSR in the Japanese population identified 2 hit SNPs of rs1691483 and rs1694933 on chromosome 3^22^, both of which were not present on the gene chip used in this study. However, these 2 SNPs were about 40000 bp next to our hit SNP of rs1032507, and the physical adjacency suggested strong linkage disequilibrium as all pairwise r^2^ among the 3 SNPs are over 0.96 in the 1000 Genome phase 3 datasets^27^. Therefore, our novel discovery of rs1032507 is in agreement with those 2 SNPs found in the Japanese population. However, it is noteworthy that the Japanese study reported a much lower PSR prevalence (3.2%) and failed to identify the replicable hit of rs10427255. Importantly, another large-size GWAS on PSR using European-descendent individuals^3^ identified rs1533426 at the 2q22.3 region (~6500 bp next to our hit of rs10427255) and rs1146751 at the 3p12.1 region (~22000 bp next to our hit of rs1032507), so results from that study and ours reproducibly map the association of PSR to the same genomic loci. In summary, the common overlap among hit SNPs from several GWAS on populations of different genetic background strongly suggested that the PSR phenotype was not ethnicity-specific but instead was inherited across ethnicities by displaying strong association with 2 independent genetic loci (2q22.3 and 3p12.1), thus substantiating the polygenic nature of PSR.

With the two genetic markers available, the biology underlying the PSR phenotype remained mysterious, and even the previous report of autosomal dominant inheritance was not conclusive^28^. A particularly interesting observation was that the variant of rs1032507 is adjacently upstream of the coding region of *CADM2*. The protein encoded by this gene is a member of the synaptic cell adhesion molecule 1 family, and plays key functions in synapse adhesion and organization. This may provide some very first preliminary links and clues for the mechanistic association between genetics and PSR.

Finally, this project demonstrated the power of the participant-driven and self-reporting phenotypic characterization. While traditional research generally avoids directly questioning sample individuals for subjective information but instead acquires phenotypic data through presumably more objective methods (e.g. physical examination, etiological analysis, medical record, etc.), certain phenotypes are either difficult to be accurately measured (PSR as an example) or tasked with just scant resources to recruit a study sample of large enough size (especially a challenge in regions or countries of large areas and less developed economies). The research model by recruiting voluntary participants to actively self-report phenotypes-of-interest and economically genotyping their SNPs has proven to be a viable strategy for conducting large-scale genotype-phenotype GWAS explorations for many largely unknown traits or diseases. We have demonstrated that such a research model is applicable to China and believe it will be continuously productive in discovering novel genetic associations with more phenotypes in future.

## Methods

### Subjects and phenotype

A total of 3519 individuals (all Chinese citizens) voluntarily participated in the project studying the association between genetic variants and PSR phenotype. All participants filled a web-based survey, designed by Chengdu 23Mofang Biotechnology Co., Ltd. (or the 23Mofang Inc.), with a single-choice question of “in most cases, when you enter from a dark or dim environment suddenly into a bright environment, would you: A. sneeze, B. be a little responsive but do not sneeze, and C. have no response”, so as to self-report the binary PSR phenotype (A as PSR-positive, B and C as PSR-negative). Additional personal information such as the sex and age of the participants were also collected in the survey for potential use as covariates in data analysis.

### Genotyping

All participants contributed 2 ml of their saliva into sample tubes. Back in laboratory, DNA was purified from the saliva, and was prepared for genotyping on a high-throughput gene chip based on the Axiom Precision Medicine Research Array (Affymetrix) with a GeneTitan Multi-Channel Instrument platform (Thermo Fisher Scientific). The gene chip covers almost nine hundred thousand SNPs spanning 22 autosomes, 2 sex chromosomes, and the mitochondrial genome (Fig. S1).

### Ethics

All participants have electronically signed the Informed Consent Form to allow their survey responses and genotypic information for the use of research purposes only. In observance of the Regulatory Articles of Human Genetic Resources in China: individual data privacy was strictly protected; all genotyping and data analysis were completed in China; linked phenotype and genotype data of participants were not submitted to protect individual genetic information, and only GWAS summary results were reported in the perspective of population genetics. The study design and research proposal were approved respectively by the Research Ethics Committee at West China School of Public Health of Sichuan University and the 23Mofang Inc. Methods were performed in accordance with the relevant guidelines and regulations.

### Data quality check and pre-processing

Raw dataset of genetic variation (3519 individuals × 819427 SNPs) underwent a series of pre-processing quality checks at both the individual and the SNP levels (details in Table S1). To remove cryptic relatedness, identity-by-descent (IBD) analysis was performed on the subset of SNPs in linkage equilibrium by sliding DNA with a 500000 base-pair (bp) window and iteratively removing adjacent SNPs exceeding a linkage disequilibrium threshold, and individuals were sequentially filtered by comparing IBD coefficients to a kinship threshold. A test of the Hardy-Weinberg equilibrium (HWE) was applied to exclude potential population substructure or genotyping errors. Principal component analysis (PCA) was performed on SNPs in linkage equilibrium, and the principal components (PCs) were used in both the ancestry check and the adjustment of remaining genetic substructure in statistical models. A final dataset of 3417 individuals × 419093 SNPs, together with the age, sex, and appropriate number of PCs, was prepared for GWAS analysis.

### Statistical methods and data analysis

Given the binary PSR phenotype as dependent variable, logistic regression was chosen for model fitting. For independent variables of SNPs, each individual was in one of three genotypes (AA, AB, BB) for a single SNP, with A and B being the corresponding minor allele and reference allele. In the additive effect model, genotypes were coded as the number of minor alleles present; in the dominant effect model, genotypes containing one or two copies of the minor allele were coded as 1; in the recessive effect model, genotypes containing two copies of the minor allele were coded as 1. Demographic variables of age and sex were included in models as covariates. Additionally, a sufficient number of PCs were also included in statistical models as covariates. P value associated with the null-hypothesis significance test for the coefficient of each SNP was converted to -log_10_(P) for evaluation, and a total of 419093 SNPs were individually tested. A stringent Bonferroni corrected threshold of α (0.05) adjusted by 1 million that results in a genome-wide significance level of P = 5 × 10^−8^, or −log_10_(P) = 7.30, was used exclusively for screening hit SNPs. A less stringent candidate threshold of α (0.05) adjusted by 1 × 10^4^ that results in a suggestive significance level of P = 5 × 10^−6^, or −log_10_(P) = 5.30, was used only in data visualization. All data analysis was conducted in version 3.4.2 of the R statistical environment (R core team, 2017)^29^, data visualization was mainly based on ggplot2^30^ and extended packages, and parallel computing in GWAS was run on a Windows computer with an Intel CPU (2.50 GHz, dual-core, 4 logic units). R code scripts used for GWAS and relevant data analysis/visualization were available upon reasonable contact with the corresponding author.

## Supplementary information

Supplementary figures and tables
