# Supplementary material for: A genome-wide association study on photic sneeze reflex in the Chinese population
Source: Sci Rep. 2019 Mar 21;9:4993. doi: 10.1038/s41598-019-41551-0 (PMC6428856; doi:10.1038/s41598-019-41551-0)
Supplement: Supplementary file 1 — Supplementary figures and tables [file 41598_2019_41551_MOESM1_ESM.pdf]

A genome-wide association study on photic sneeze reflex in the Chinese population

Mengqiao Wang[#]*, Xinghan Sun[#], Yang Shi, Xiaojun Song, Hao Mi

[#] these authors contributed equally
* corresponding author

Supplementary Figures

Figure S1

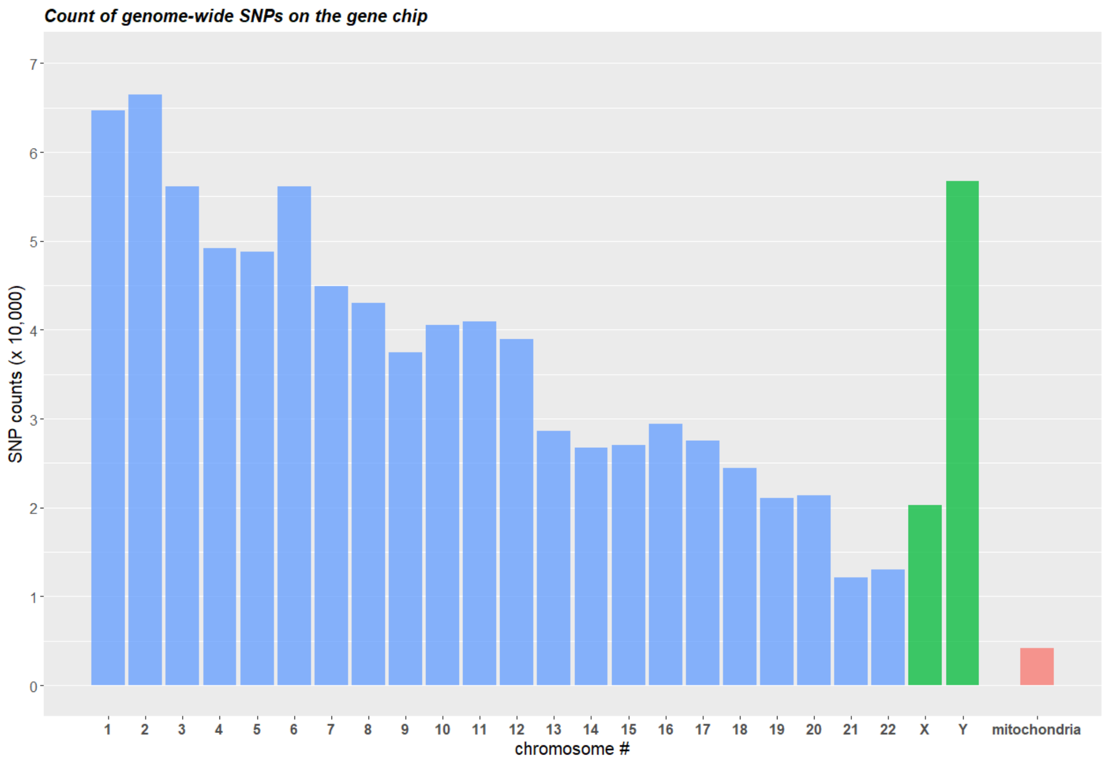

Figure S2

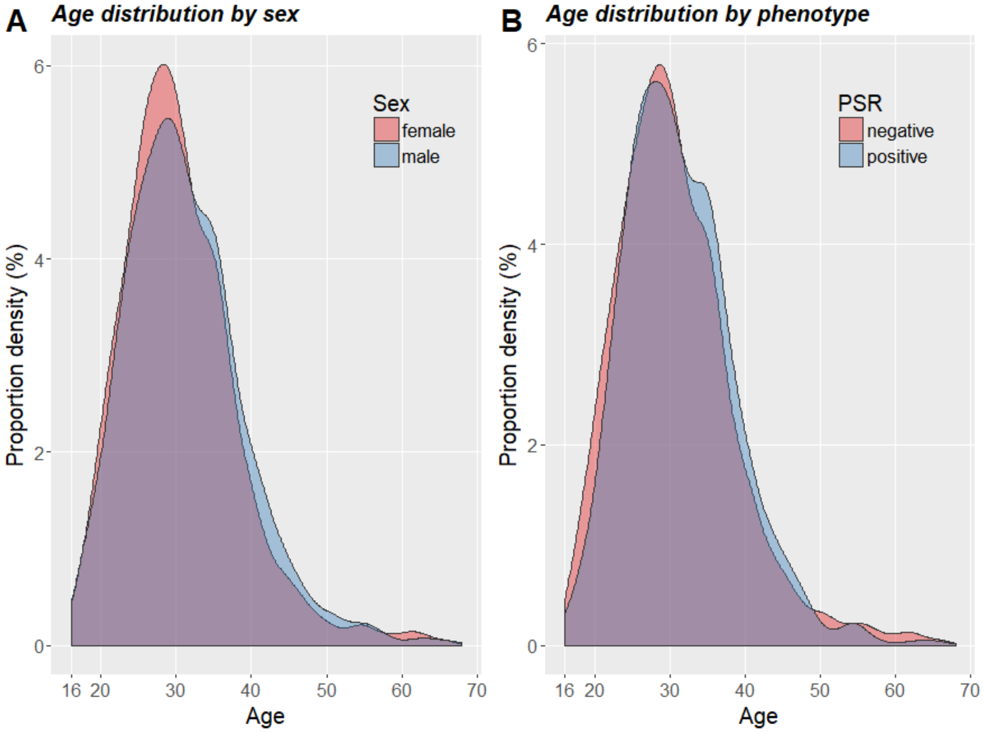
A
Age distribution by sex
Sex
female
male
Proportion density (%)
0
2
4
6
16 20 30 40 50 60 70
Age
B
Age distribution by phenotype
PSR
negative
positive
Proportion density (%)
0
2
4
6
16 20 30 40 50 60 70
Age

Figure S3

A

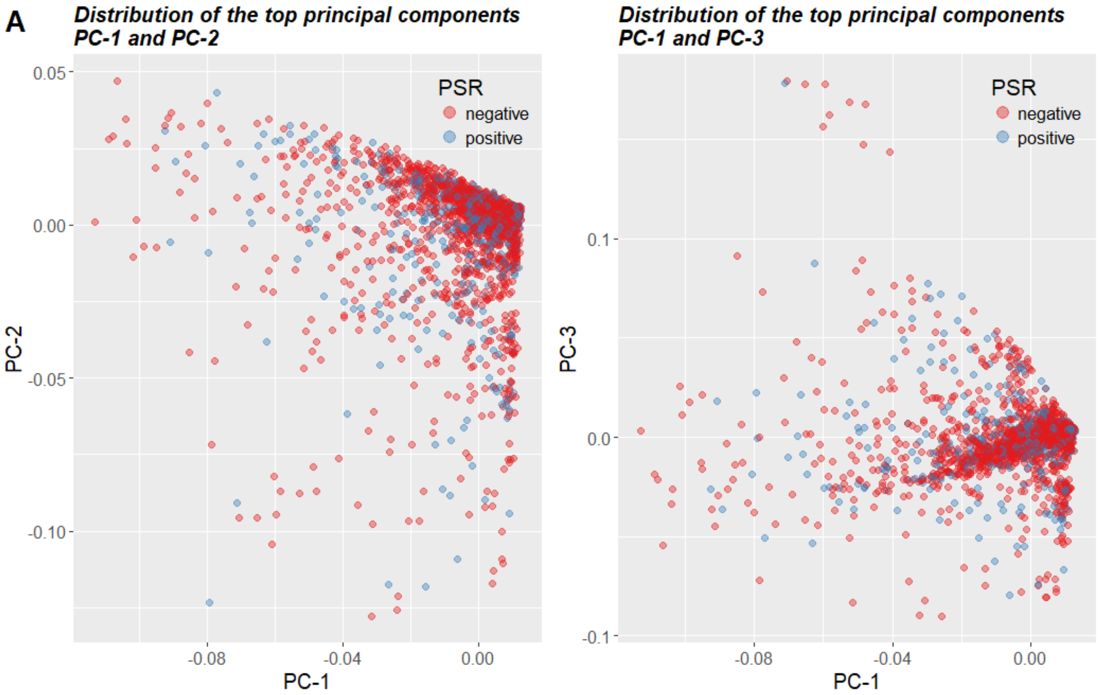

Distribution of the top principal components PC-1 and PC-2
PSR
negative
positive
PC-2
PC-1
0.05
0.00
-0.05
-0.10
-0.08
-0.04
0.00
Distribution of the top principal components PC-1 and PC-3
PC-3
0.1
0.0
-0.1

B

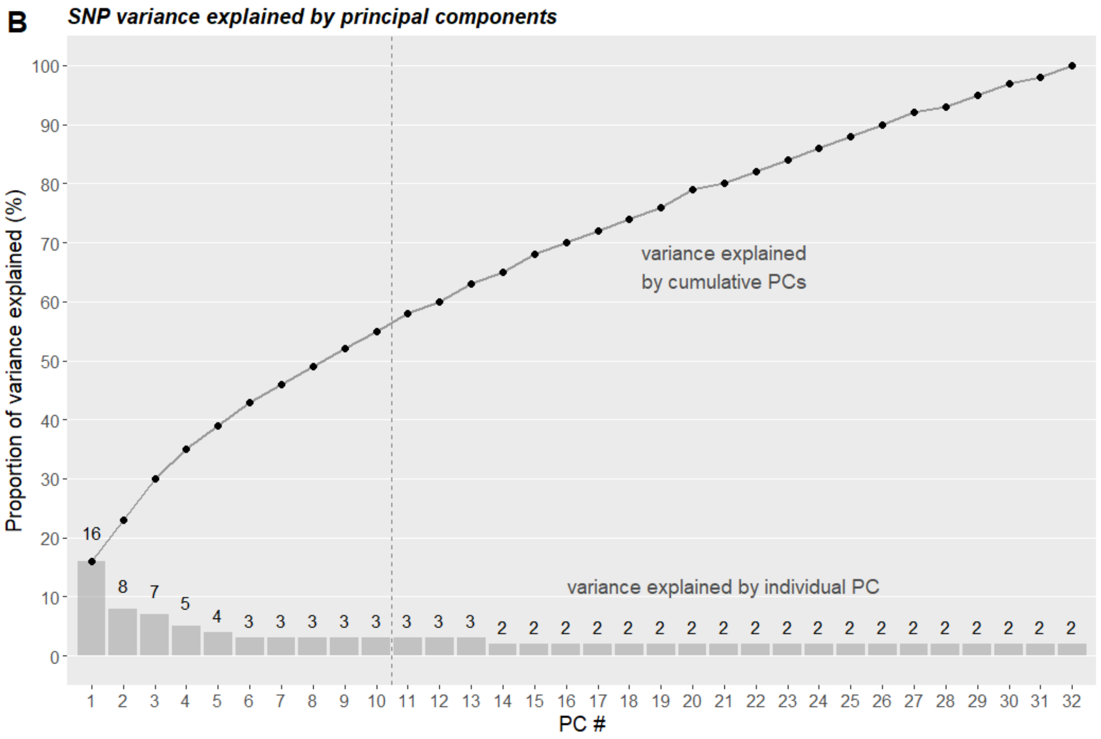

SNP variance explained by principal components
Proportion of variance explained (%)
variance explained by cumulative PCs
variance explained by individual PC
PC #

Figure S4

A

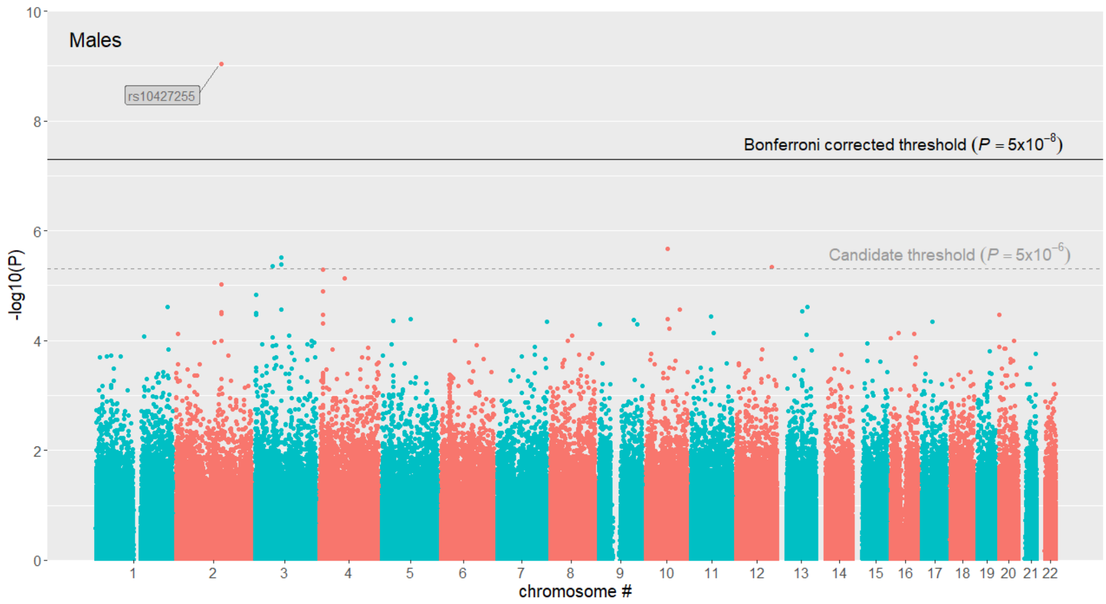
Males
rs10427255
Bonferroni corrected threshold ($P = 5x10^{-8}$)
Candidate threshold ($P = 5x10^{-6}$)
-log10(P)
0
2
4
6
8
10
1 2 3 4 5 6 7 8 9 10 11 12 13 14 15 16 17 18 19 20 21 22
chromosome #

B

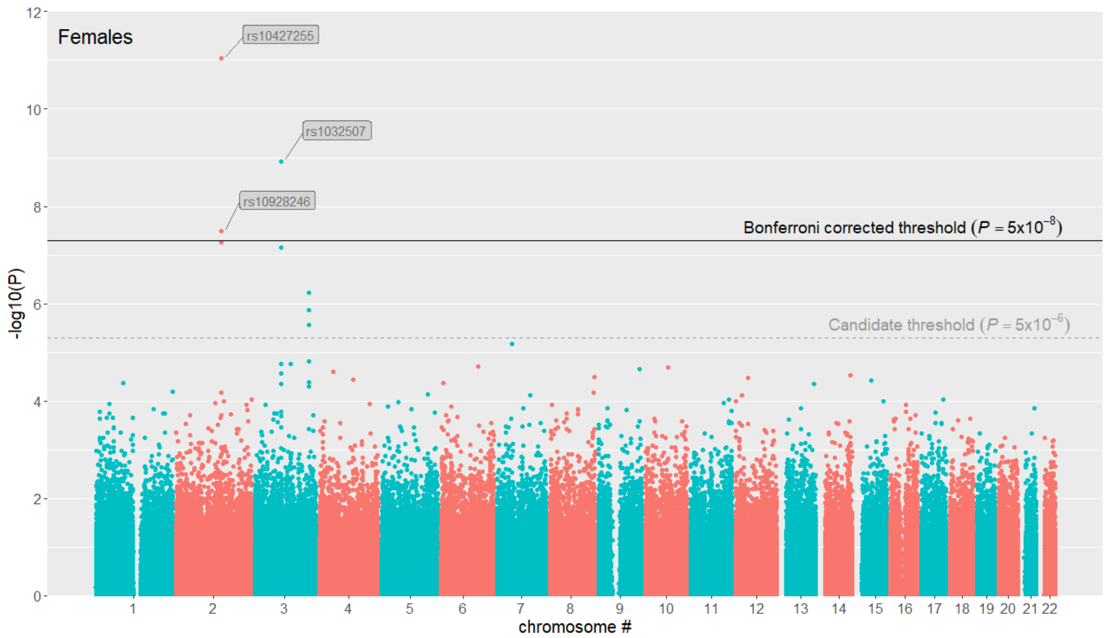
Females
rs10427255
rs1032507
rs10928246
Bonferroni corrected threshold ($P = 5x10^{-8}$)
Candidate threshold ($P = 5x10^{-6}$)
-log10(P)
0
2
4
6
8
10
12
1 2 3 4 5 6 7 8 9 10 11 12 13 14 15 16 17 18 19 20 21 22
chromosome #

Figure S5

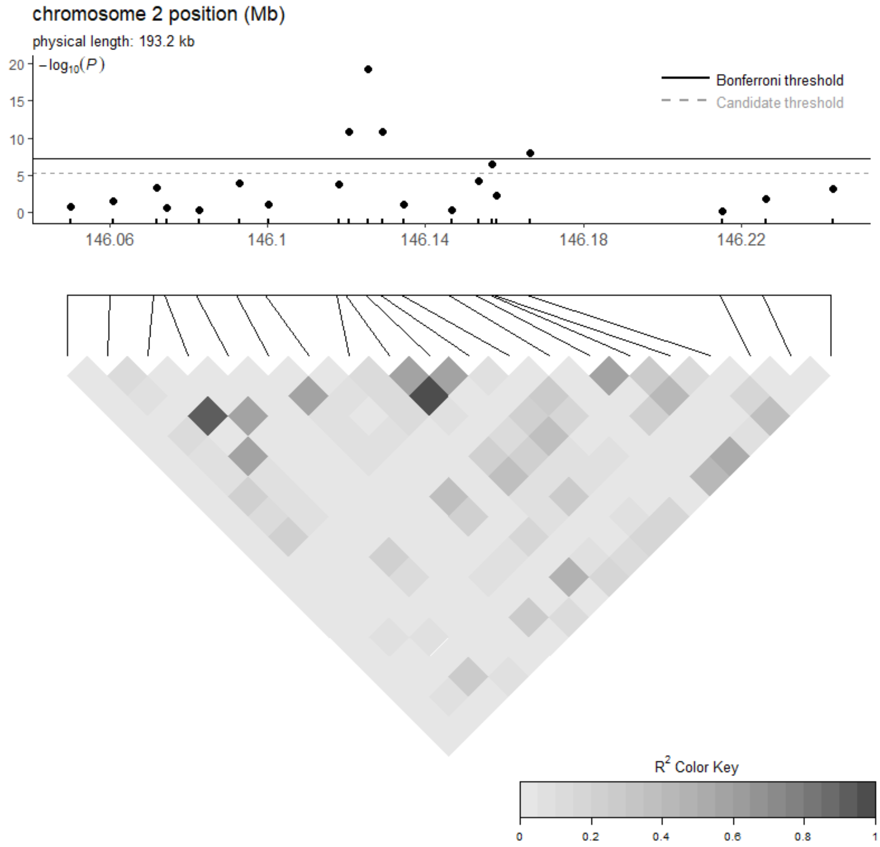

chromosome 2 position (Mb)
physical length: 193.2 kb
$-\log_{10}(P)$
20
15
10
5
0
Bonferroni threshold
Candidate threshold
146.06
146.1
146.14
146.18
146.22
R^2 Color Key
0
0.2
0.4
0.6
0.8
1

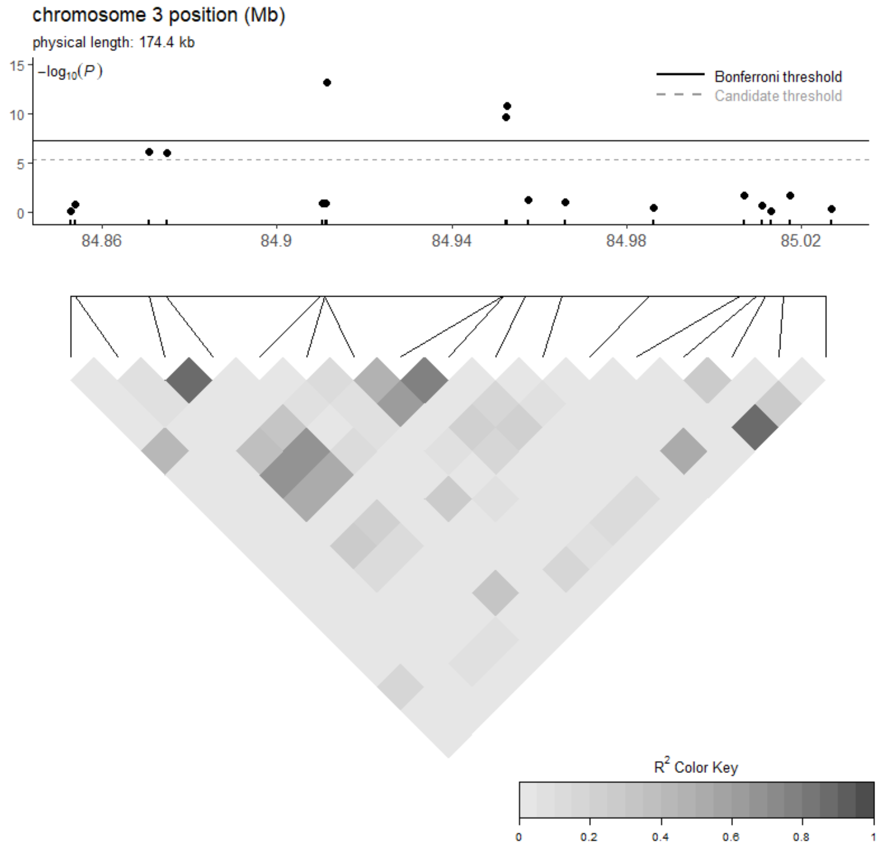

chromosome 3 position (Mb)
physical length: 174.4 kb
$-\log_{10}(P)$
15
10
5
0
Bonferroni threshold
Candidate threshold
84.86
84.9
84.94
84.98
85.02
R^2 Color Key
0
0.2
0.4
0.6
0.8
1

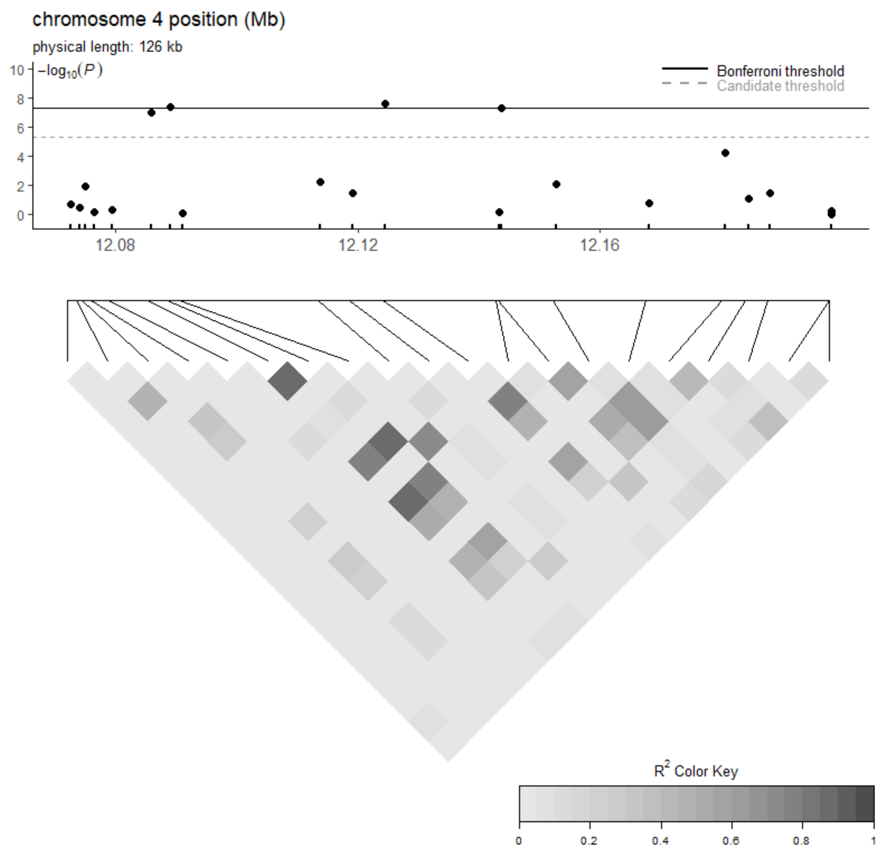
chromosome 4 position (Mb)
physical length: 126 kb
−log10(P)
Bonferroni threshold
Candidate threshold
10
8
6
4
2
0
12.08
12.12
12.16
R2 Color Key
0
0.2
0.4
0.6
0.8
1

Figure S6

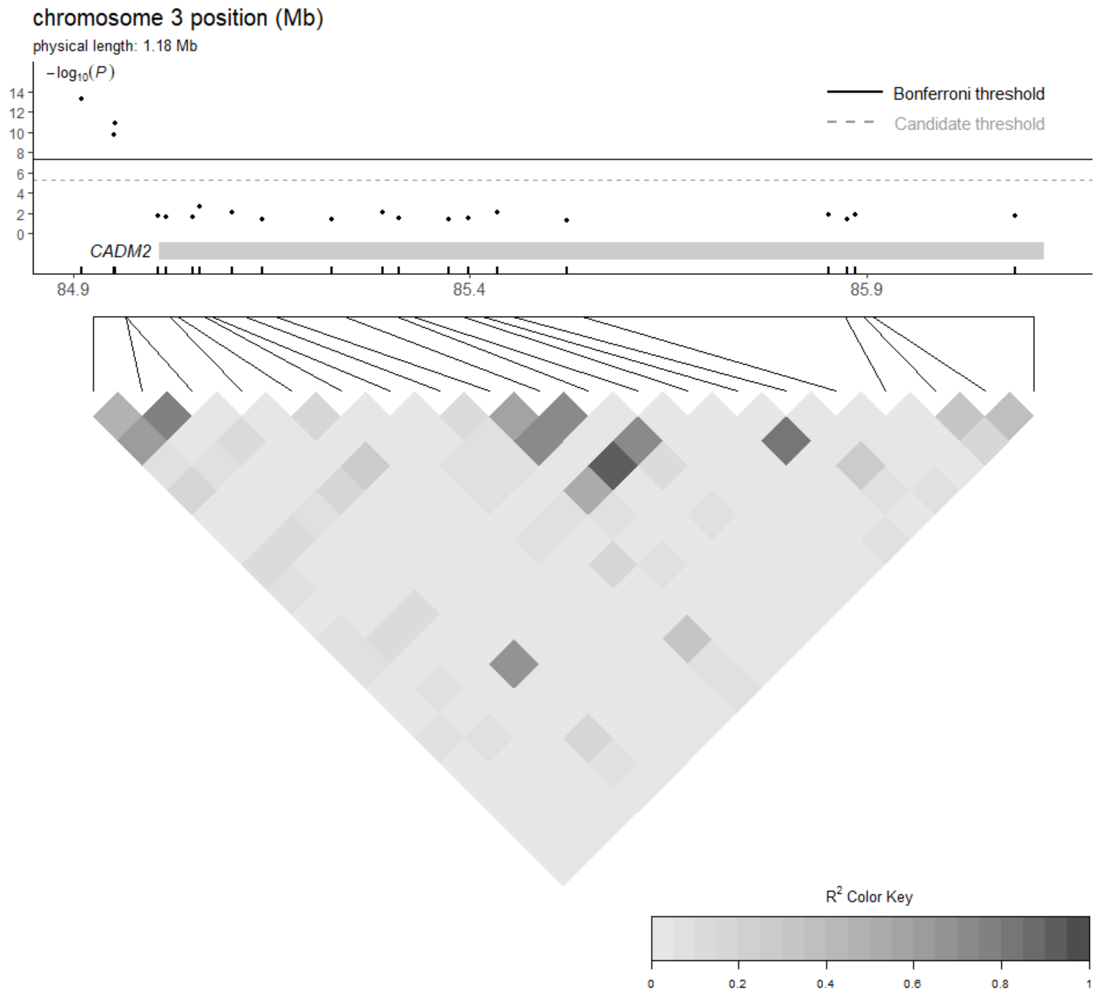

chromosome 3 position (Mb)
physical length: 1.18 Mb
−log10(P)
14
12
10
8
6
4
2
0
Bonferroni threshold
Candidate threshold
CADM2
84.9
85.4
85.9
R2 Color Key
0
0.2
0.4
0.6
0.8
1

Figure S7

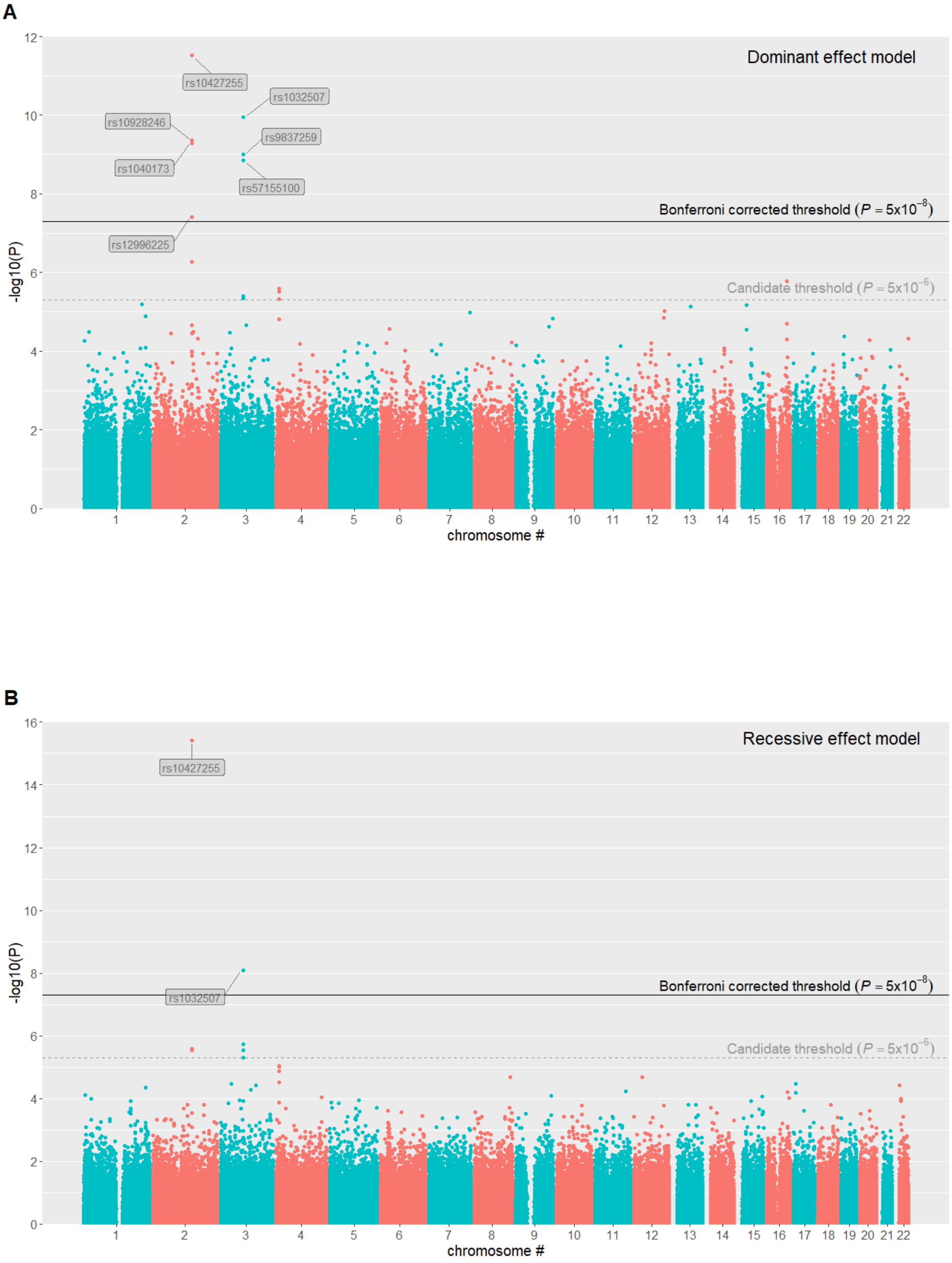
A
Dominant effect model
rs10427255
rs1032507
rs10928246
rs9837259
rs1040173
rs57155100
rs12996225
Bonferroni corrected threshold ($P = 5x10^{-8}$)
Candidate threshold ($P = 5x10^{-6}$)
-log10(P)
chromosome #
B
Recessive effect model
rs10427255
rs1032507
Bonferroni corrected threshold ($P = 5x10^{-8}$)
Candidate threshold ($P = 5x10^{-6}$)
-log10(P)
chromosome #

Figure S8

A

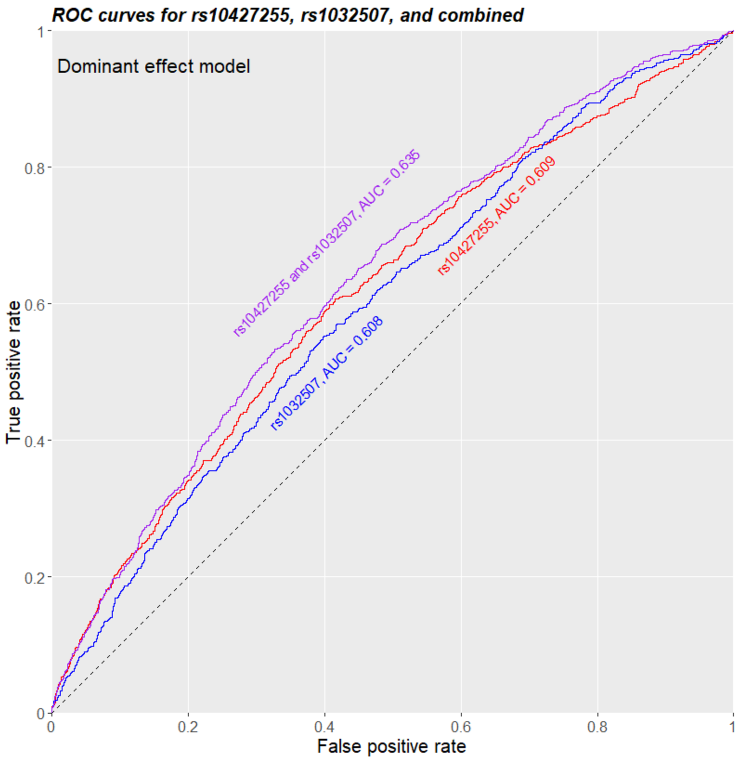
ROC curves for rs10427255, rs1032507, and combined
Dominant effect model
rs10427255 and rs1032507, AUC = 0.635
rs10427255, AUC = 0.609
rs1032507, AUC = 0.608
True positive rate
False positive rate
0
0.2
0.4
0.6
0.8
1

B

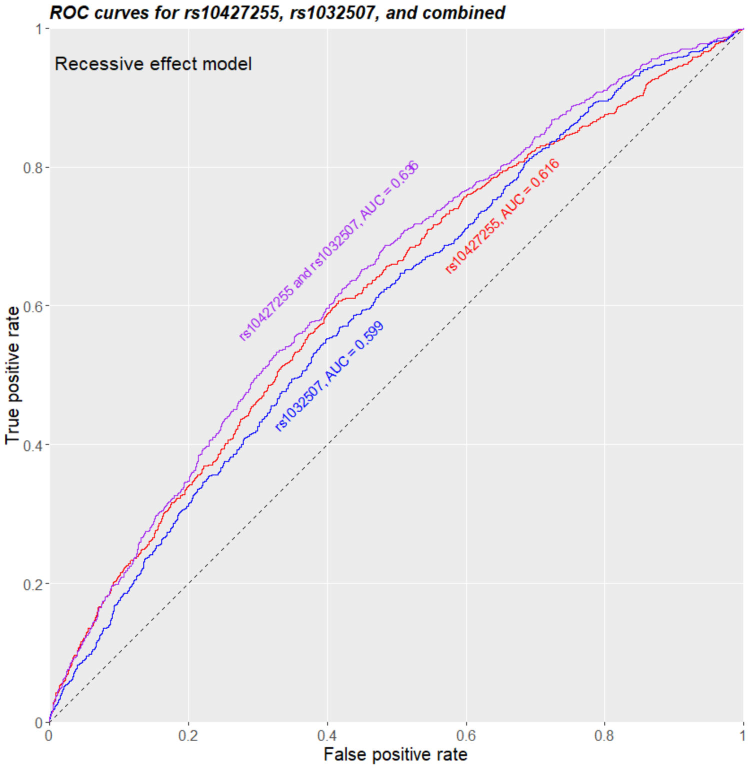
ROC curves for rs10427255, rs1032507, and combined
Recessive effect model
rs10427255 and rs1032507, AUC = 0.636
rs10427255, AUC = 0.616
rs1032507, AUC = 0.599
True positive rate
False positive rate
0
0.2
0.4
0.6
0.8
1

Supplementary Figure Legends

Figure S1. Distribution for the count of SNPs on the gene chip used for genotyping.

Figure S2. Age distribution by sex (A) or by phenotype (B) of sample individuals.

Figure S3. Principal component analysis of SNPs. PC-1, PC-2, and PC-3 represented the top 3 principal components (PCs) explaining the highest portion of genetic variation. A. Scatter plots of PC-1 *vs.* PC-2, and PC-1 *vs.* PC-3, displayed the clustering of the surveyed individuals as a homogenous sample. B. Individual and cumulative proportion of variance explained by the corresponding PCs. The top 10 PCs cumulatively explained over 55% of total variance.

Figure S4. Sex-separated analysis: Manhattan plot of GWAS based on an additive effect model for males (A) or for females (B). Bonferroni corrected threshold and candidate threshold respectively correspond to 7.30 and 5.30 with regard to $-\log_{10}(P)$.

Figure S5. Zoomed-in regional association plot of 3 hit SNP regions.

Figure S6. Association plot of hit SNPs on chromosome 3 to SNPs within their nearest downstream gene of *CADM2*.

Figure S7. Manhattan plot of GWAS based on the dominant effect model (A) or the recessive effect model (B).

Figure S8. ROC curves for predicting PSR phenotype using rs10427255 alone (red), rs1032507 alone (blue), or rs10427255 and rs1032507 combined (purple), based on the dominant effect model (A) or the recessive effect model (B).

Supplementary Tables

Table S1.

Pre-processing steps to assemble the final dataset for GWAS

Step	Pre-processing	Sample	SNP	Note
1	initial	3519	819427	
2	sex check	3519	819427	check the match between self-reported sex and gene-chip-revealed sex
3	age check	3468	819427	exclusion of 51 individuals with self-reported age outside the [16, 70] range
4	SNPs on 22 autosomes	3468	738243	exclusion of 81184 non-autosome SNPs
5	SNP-level filtering	3468	430822	exclusion of 307421 SNPs due to low call rate (37711, threshold = 0.95) or low minor allele frequency (233609, threshold = 0.01) or both (36101)
6	sample-level filtering	3432	430822	exclusion of 36 individuals due to low call rate (14, threshold = 0.95) or high inbreeding coefficient (20, threshold = 0.1) or both (2)
7	kinship filtering	3417	430822	exclusion of 15 individuals due to cryptic relatedness using identity-by-descent analysis (threshold = 0.1)
8	ancestry filtering	3417	430822	no individual excluded given the largely homogeneous Chinese ancestry in PCA
9	HWE filtering	3417	419093	exclusion of 11729 SNPs due to the rejection of Hardy-Weinberg Equilibrium at adjusted α of 1×10^{-6}
